# Supplementary material for: Graph identification of proteins in tomograms (GRIP‐Tomo)
Source: Protein Sci. 2023 Jan 1;32(1):e4538. doi: 10.1002/pro.4538 (PMC9798246; doi:10.1002/pro.4538)
Supplement: Supplementary file 1 — Figure S1. (a) The ribbon representation of space‐filling Ankyrin repeats (4RLY) (R1‐R9) model. The graph network representation of this structure at (b) dcut = 8 Å and (c) dcut = 9 Å. The highlighted nodes (residues) on (b) were located on the structural helices whereas those in (c) were located on the binding segments of the chain across the helical R repeats. The structural pairs corresponding to the high‐BC nodes on the graph with dcut = 9 Å are colored in (a), capturing a higher‐order complexity across repeated structural helices within a single domain. Figure S2. (a, c) The ribbon representation of a space‐filling four‐helix bundle protein (dimer of dimer, 3VJF). The graph network representation of (b, c) protein structures at (b) dcut = 7 Å and (d) dcut = 8 Å, respectively. (d) identified four residues with extremely high BC scores. The amino residues with the two highest scores were copies of PHE85, located on both the A and B chains. The third and fourth highest scoring residues were copies of ASN34 – another pair of identical aminos on the A and B chains in (c). 8 Å is the best choice of cutoff distance for identifying the cross‐helix and cross‐chain interaction, indicating the structural importance of the residues in the higher‐order complexity of dimer of dimer. Figure S3. (a) The ribbon representation of the space filling hemoglobin (tetramer, 1A3N). The graph network representation of the tetramer at (b) dcut = 7 Å, (c) dcut = 8 Å, and (d) dcut = 9 Å. The graph with dcut = 8 Å contained the most distinct cluster of nodes (residues) with high BC. These clusters contained residues (colored in (a)) on multiple chains within proximity of each other, indicating cross‐chain interaction. Figure S4. (a) The ribbon representation of the Apoferritin complex, containing 24 units of the same chain (2W0O) in an octahedral symmetry. The graph network representation Apoferritin at (b) dcut = 7 Å, (c) dcut = 8 Å, and (d) dcut = 9 Å. These nodes on the graph with (c) d [file PRO-32-e4538-s001.docx]

Supplementary Information

Graph identification of proteins in tomograms (GRIP-Tomo)

August George^1,2^, Doo Nam Kim^3^, Trevor Moser^1^, Ian T. Gildea^1^, James E. Evans^*1,4^, Margaret S. Cheung*^1,5^

^1^ Environmental Molecular Sciences Laboratory, Pacific Northwest National Laboratory, Richland, WA, 99354, USA

^2^ Department of Biomedical Engineering, Oregon Health & Science University, Portland, OR, 97239, USA

^3^ Biological Science Division, Pacific Northwest National Laboratory, Richland, WA, 99354, USA

^4^ School of Biological Sciences, Washington State University, Pullman, WA, 99164, USA

^5^ Department of Physics, University of Washington, Seattle, WA, 98195 USA

* Corresponding authors: Margaret.Cheung@PNNL.Gov, James.Evans@PNNL.Gov

1. **Supporting Materials**
   1. **Supporting Figures 1 – 7**

**
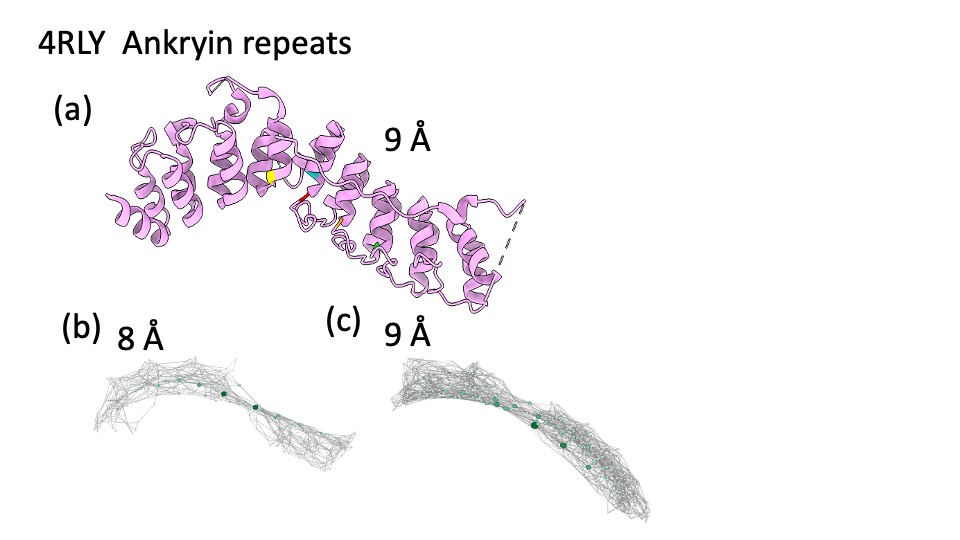
**

**Figure S1:** (a) The ribbon representation of space-filling Ankyrin repeats (4RLY) (R1-R9) model. The graph network representation of this structure at (b) d_cut_=8 Å and (c) d_cut_=9 Å. The highlighted nodes (residues) on (b) were located on the structural helices whereas those in (c) were located on the binding segments of the chain across the helical R repeats. The structural pairs corresponding to the high-BC nodes on the graph with d_cut_=9 Å are colored in (a), capturing a higher-order complexity across repeated structural helices within a single domain.


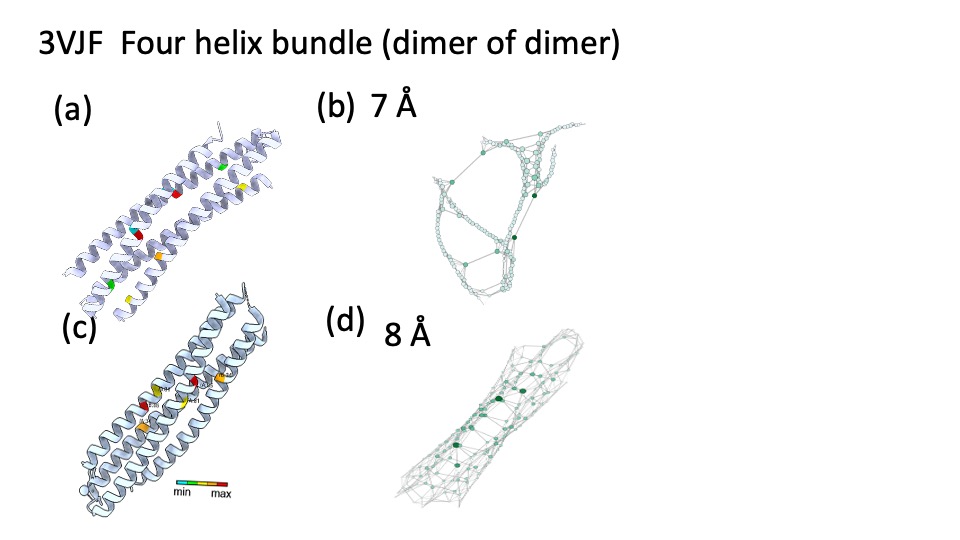


**Figure S2:** (a, c) The ribbon representation of a space-filling four-helix bundle protein (dimer of dimer, 3VJF). The graph network representation of (b, c) protein structures at (b) d_cut_ = 7 Å and (d) d_cut_ = 8 Å, respectively. (d) identified four residues with extremely high BC scores. The amino residues with the two highest scores were copies of PHE85, located on both the A and B chains. The third and fourth highest scoring residues were copies of ASN34 – another pair of identical aminos on the A and B chains in (c). 8 Å is the best choice of cutoff distance for identifying the cross-helix and cross-chain interaction, indicating the structural importance of the residues in the higher-order complexity of dimer of dimer.


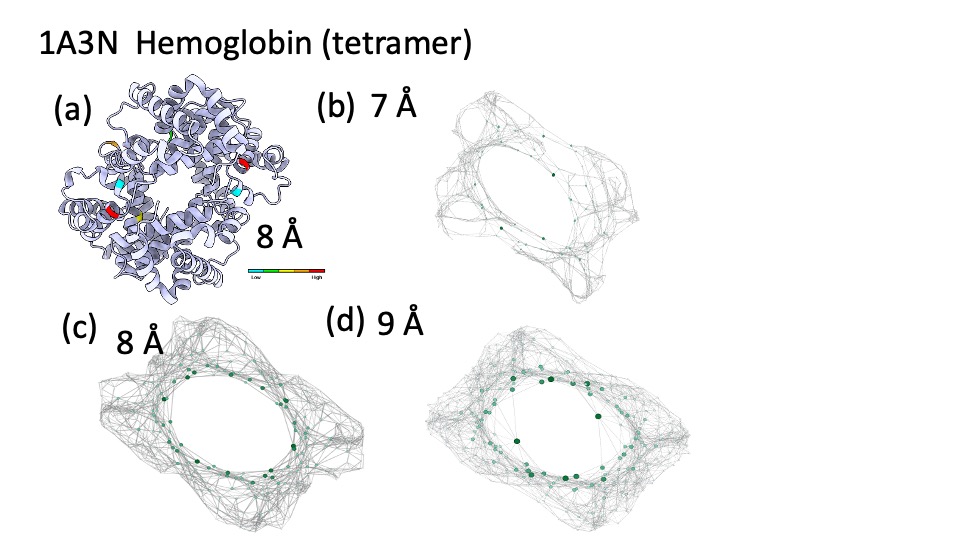


**Figure S3:** (a) The ribbon representation of the space filling hemoglobin (tetramer, 1A3N). The graph network representation of the tetramer at (b) d_cut_ = 7 Å, (c) d_cut_ = 8 Å, and (d) d_cut_ = 9 Å. The graph with d_cut_ = 8 Å contained the most distinct cluster of nodes (residues) with high BC. These clusters contained residues (colored in (a)) on multiple chains within proximity of each other, indicating cross-chain interaction.

**
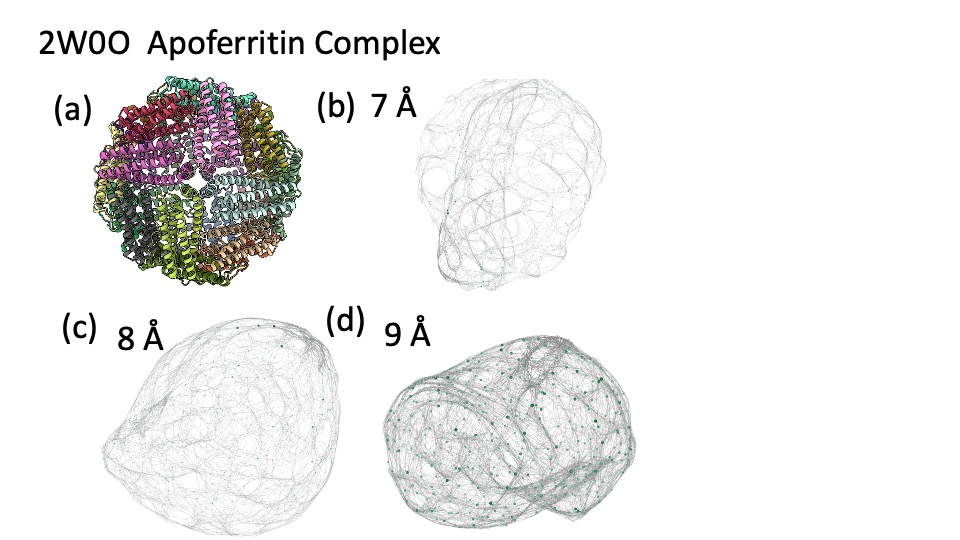
**

**Figure S4:** (a) The ribbon representation of the Apoferritin complex, containing 24 units of the same chain (2W0O) in an octahedral symmetry. The graph network representation Apoferritin at (b) d_cut_ = 7 Å, (c) d_cut_ = 8 Å, and (d) d_cut_ = 9 Å. These nodes on the graph with (c) d_cut_=8 Å become more prominent with high BC than those in (b) d_cut_=7 Å. (d) At d_cut_=9 Å, residues with high BC appeared in the center part of each chain, instead of at the vertex that captures the cross-unit interaction.


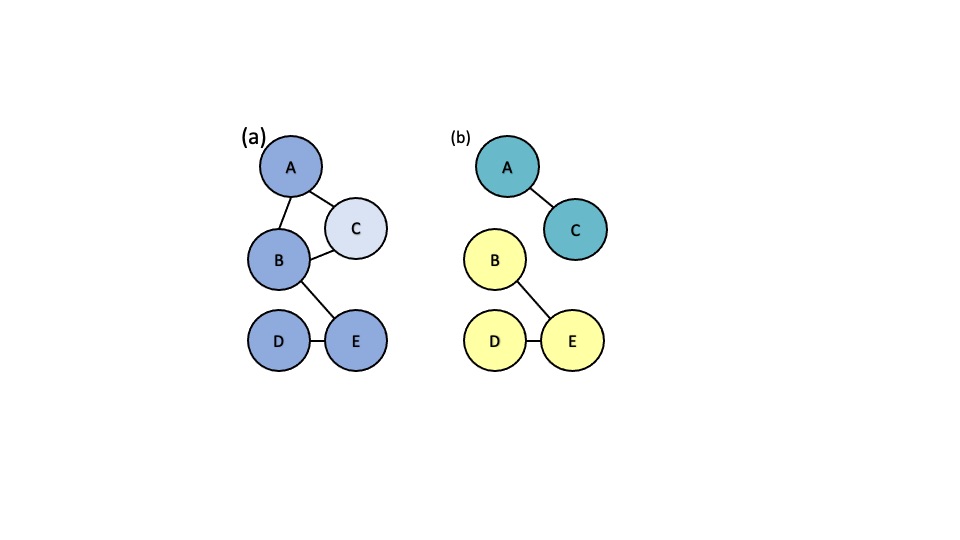


**Figure S5**: Fundamental graph properties at a node level on two network models (a) and (b). Each model comprises five nodes, V={A,B,C,D,E}. The difference between the network in (a) and the network in (b) is the set of edges connecting these nodes. Graph (a) has one triangle ({A,B,C}). While in graph (a) all nodes are connected, in graph (b) there are two separate components: {A,C} and {B,D,E}. In (a), the clique number, which is the largest subset of nodes that are all directly connected, is 3, and the largest clique is {A,B,C}. In contrast, the largest clique in graph (b) is {A,C}. The degree of a node is the number of edges connected to it. For example, in (a), the degree of node B equals 3. The shortest path between two nodes is the minimal number of edges one needs to hop over to reach from one node to the other. In graph (a), the shortest path between node D and node A is D↔E↔B↔A. Nodes that are disconnected, as in the case of D and A in (b), are said to be separated by an infinite path by definition. The betweenness of a node is the number of the shortest paths between any other nodes that go through this node. For example, in (a), the node B has the largest betweenness of 4, as the shortest path between D and A, between D and C, between E and A, and between E and C, all go through B.


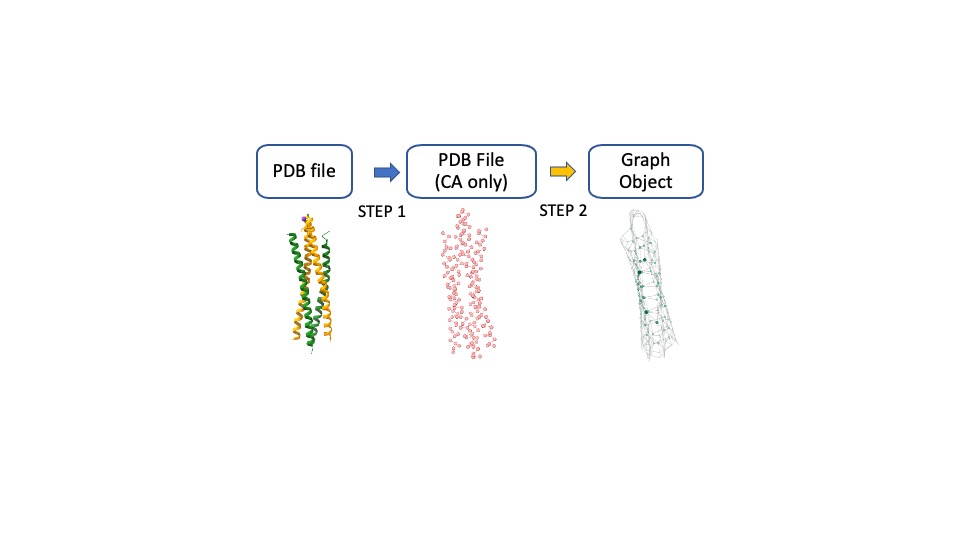


**Figure S6:** The procedure to convert a 3D protein structure (.pdb) into a graph (.gexf). In Step 1, the protein structure with an all-atomistic representation is coarse-grained to a minimalist representation with only alpha carbons. The alpha carbons are assigned as vertices in a graph. In Step 2, for each pair of graph vertices (alpha carbons) the Euclidean distance is calculated. If the distance is less than or equal to d_cut_ (ie.g. 8 Angstroms), an edge is connected between the two vertices. The resulting graph is then saved as a graph exchange XML file (.gexf). The NetworkX package of Python was used to process and export the graph for further analysis.


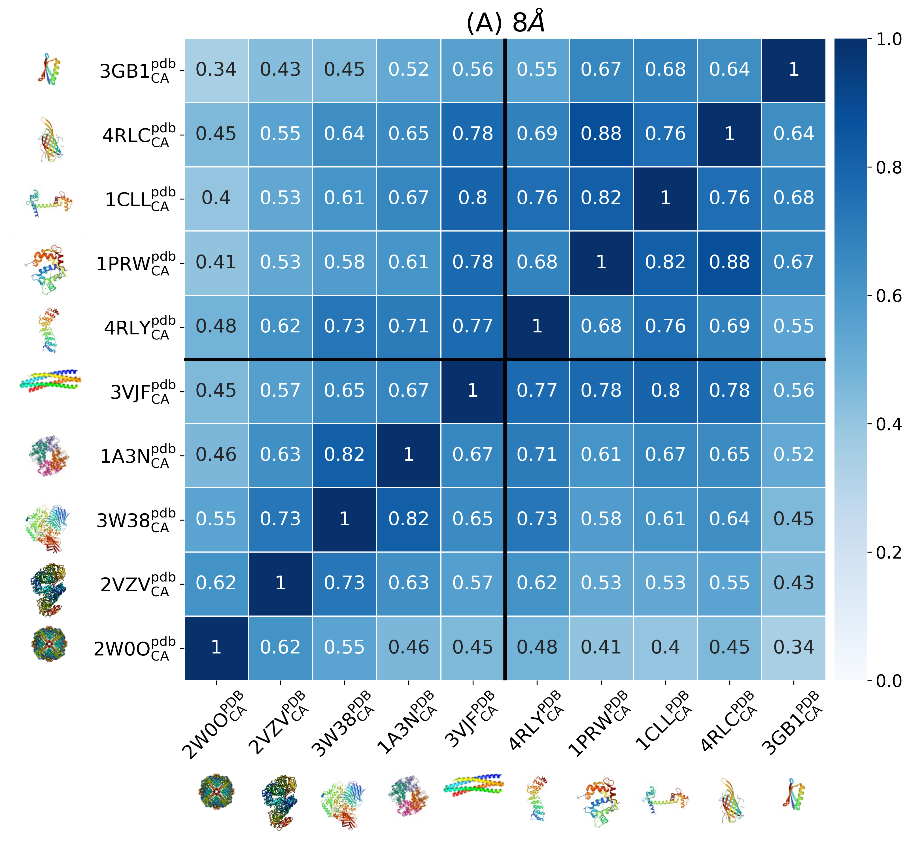

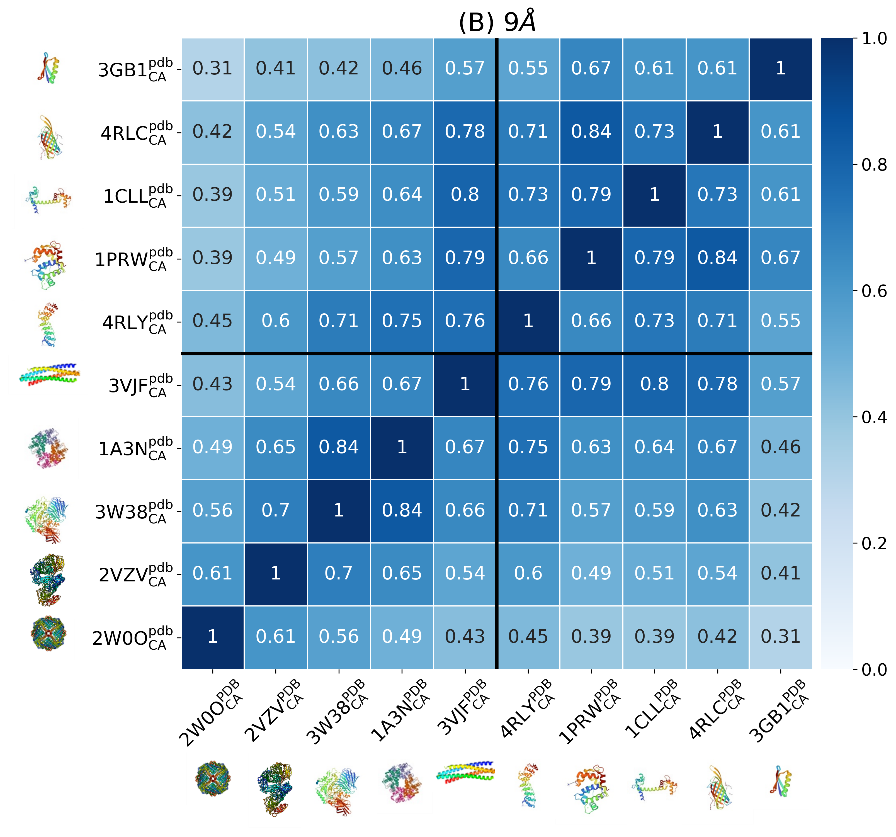


**Figure S7:** The similarity scores (χ) by comparing the Control Graphs against each other for the 10 selected protein structures or complexes using d_cut_=8Å for (a) and d_cut_= 9Å for (b). As expected, graphs from proteins of similar size and complexity tend to have the largest similarity scores – with the same graphs having the maximum similarity score of 1.
